# Supplementary material for: Development and evaluation of statistical and artificial intelligence approaches with microbial shotgun metagenomics data as an untargeted screening tool for use in food production
Source: mSystems. 2024 Oct 10;9(11):e00840-24. doi: 10.1128/msystems.00840-24 (PMC11575248; doi:10.1128/msystems.00840-24)
Supplement: Supplemental figures — Figures S1 to S4. [file msystems.00840-24-s0001.pdf]

Supplemental Figures

Development and evaluation of statistical and Artificial Intelligence approaches with microbial shotgun metagenomics data as an untargeted screening tool for use in food production

Beck et al., 2024

Block	Date	BL	OF	ABX
	5-Sep-18	1	1	
	6-Sep-18	2		1
	7-Sep-18	3		2
	8-Sep-18	4		
	9-Sep-18	5		
	10-Sep-18	6	2	
	11-Sep-18	7	3	
	12-Sep-18	8	4	
	13-Sep-18	9		3
	14-Sep-18	10		4
	15-Sep-18	11		
	16-Sep-18	12		
	17-Sep-18	13	5	
	18-Sep-18	14		
	19-Sep-18	15		5
	20-Sep-18	16	6	6
	21-Sep-18	17		7
	22-Sep-18	18	7	
	23-Sep-18	19		
	24-Sep-18	20	8	
	25-Sep-18	21		
	26-Sep-18	22		8
	27-Sep-18	23		
	28-Sep-18	24	9	
	29-Sep-18	25		9
	30-Sep-18	26		
	1-Oct-18	27	10	
	2-Oct-18	28		
	3-Oct-18	29	11	10
	4-Oct-18	30		11
	5-Oct-18	31		12
	6-Oct-18	32	12	
	7-Oct-18	33	13	

Supplemental Figure S1: Sampling Scheme. Dates were block randomized to ensure even

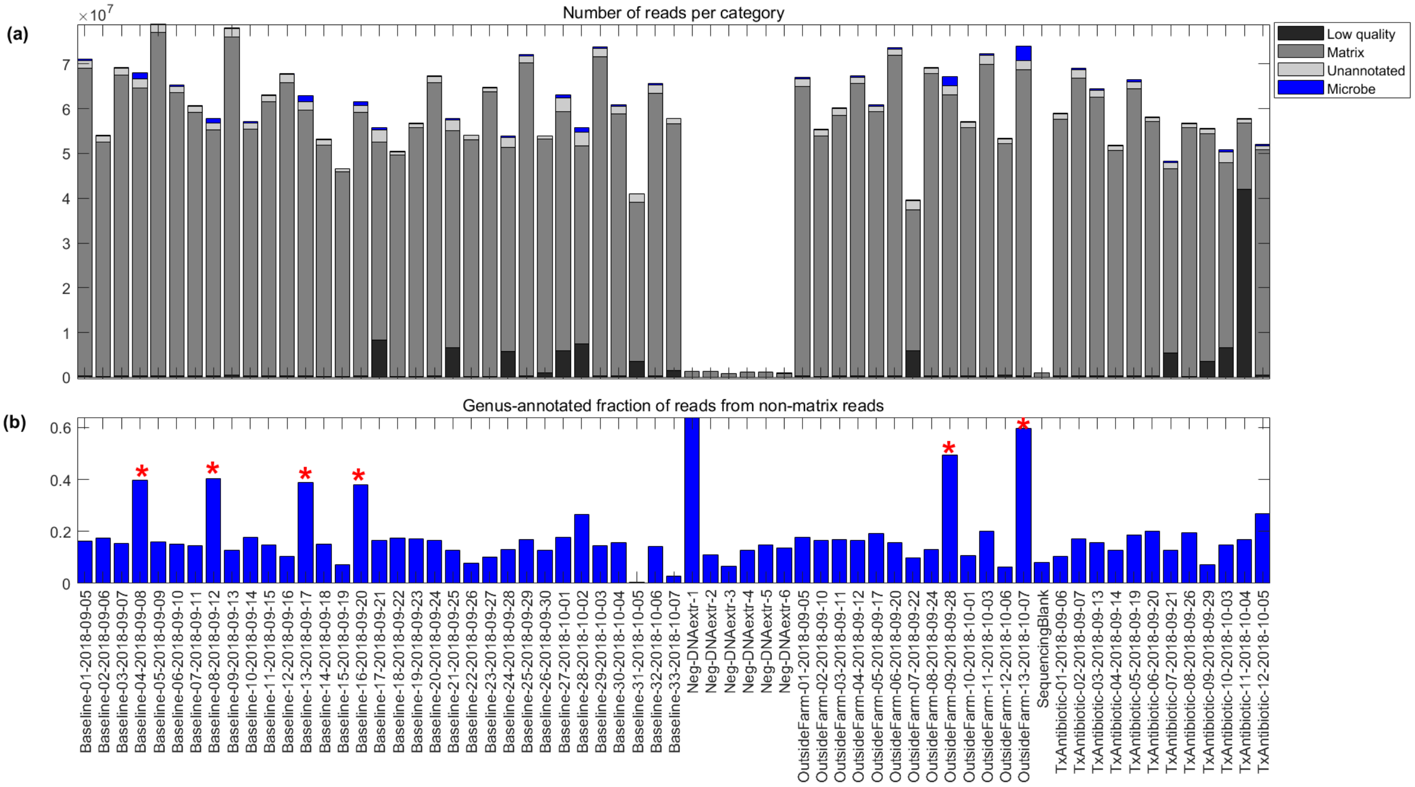

Supplemental Figure S2: Read counts per sample. **(a)** Total number of input reads per sample are shown, divided into low quality, matrix filtered, unannotated, and microbial genus-assigned (after removing contaminating genera). **(b)** The fraction of genus-annotated reads from the non-matrix reads with low-diversity outlier samples indicated with red stars.

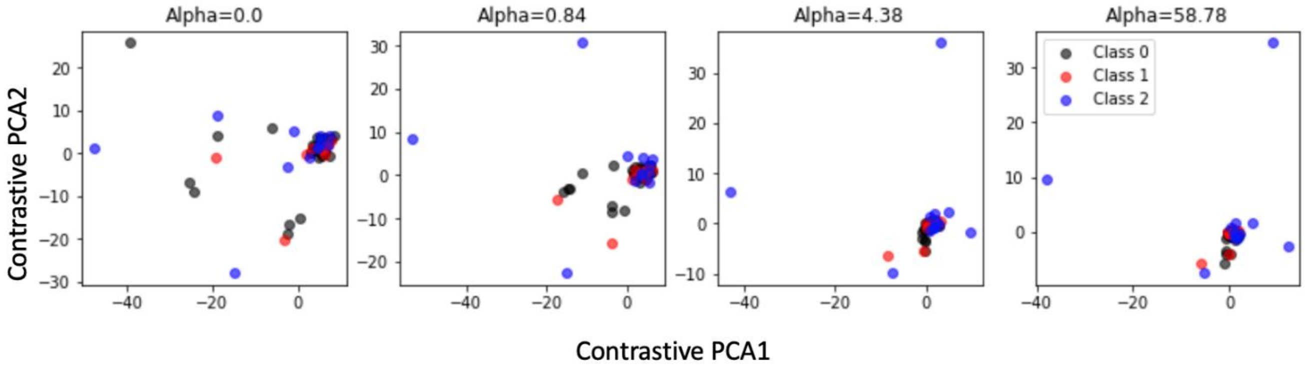

Supplemental Figure S3: Contrastive PCA results. Each box corresponds to a different value of

(a) Outside farm vs. baseline

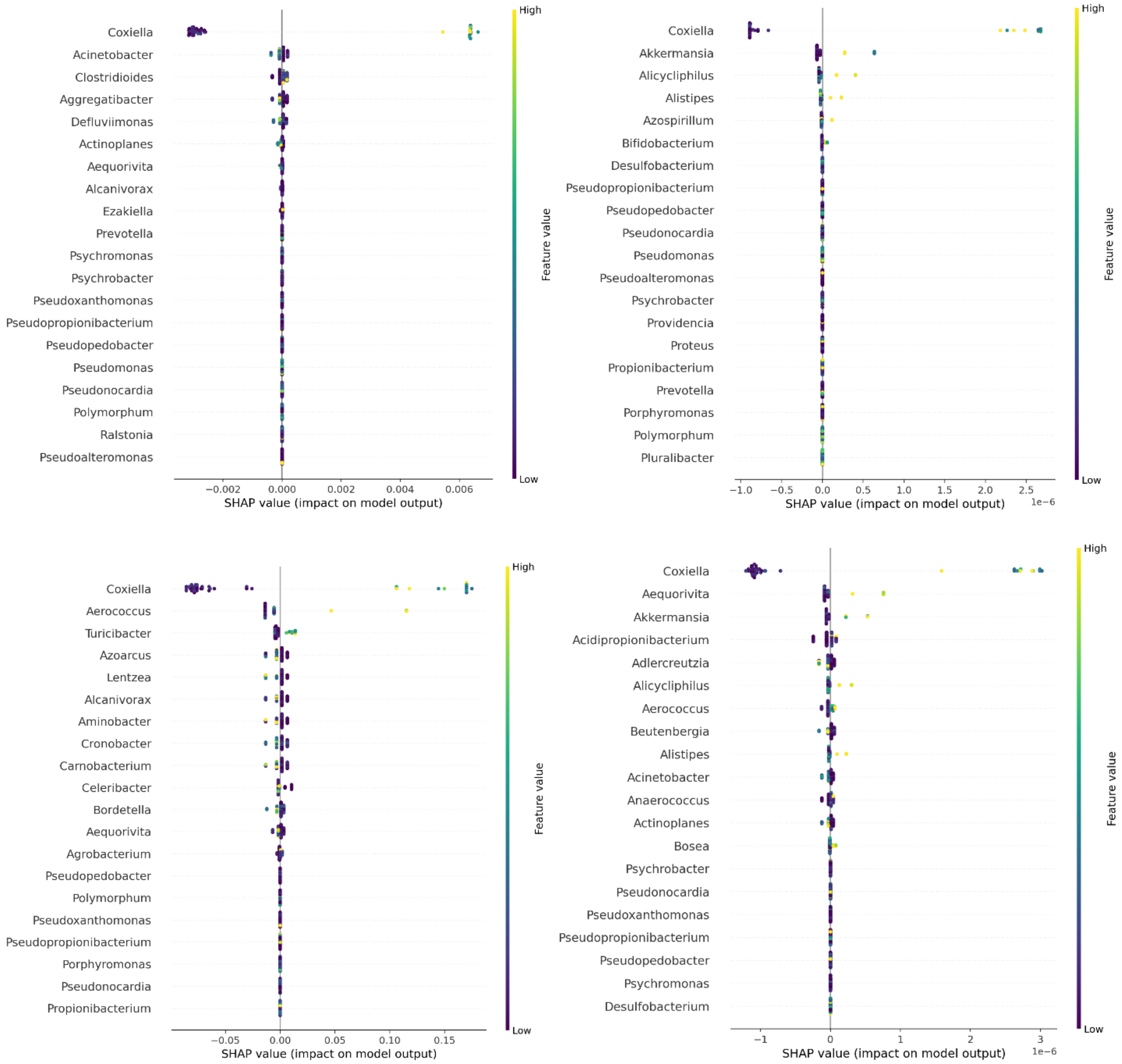

(b) Antibiotic treated vs. baseline

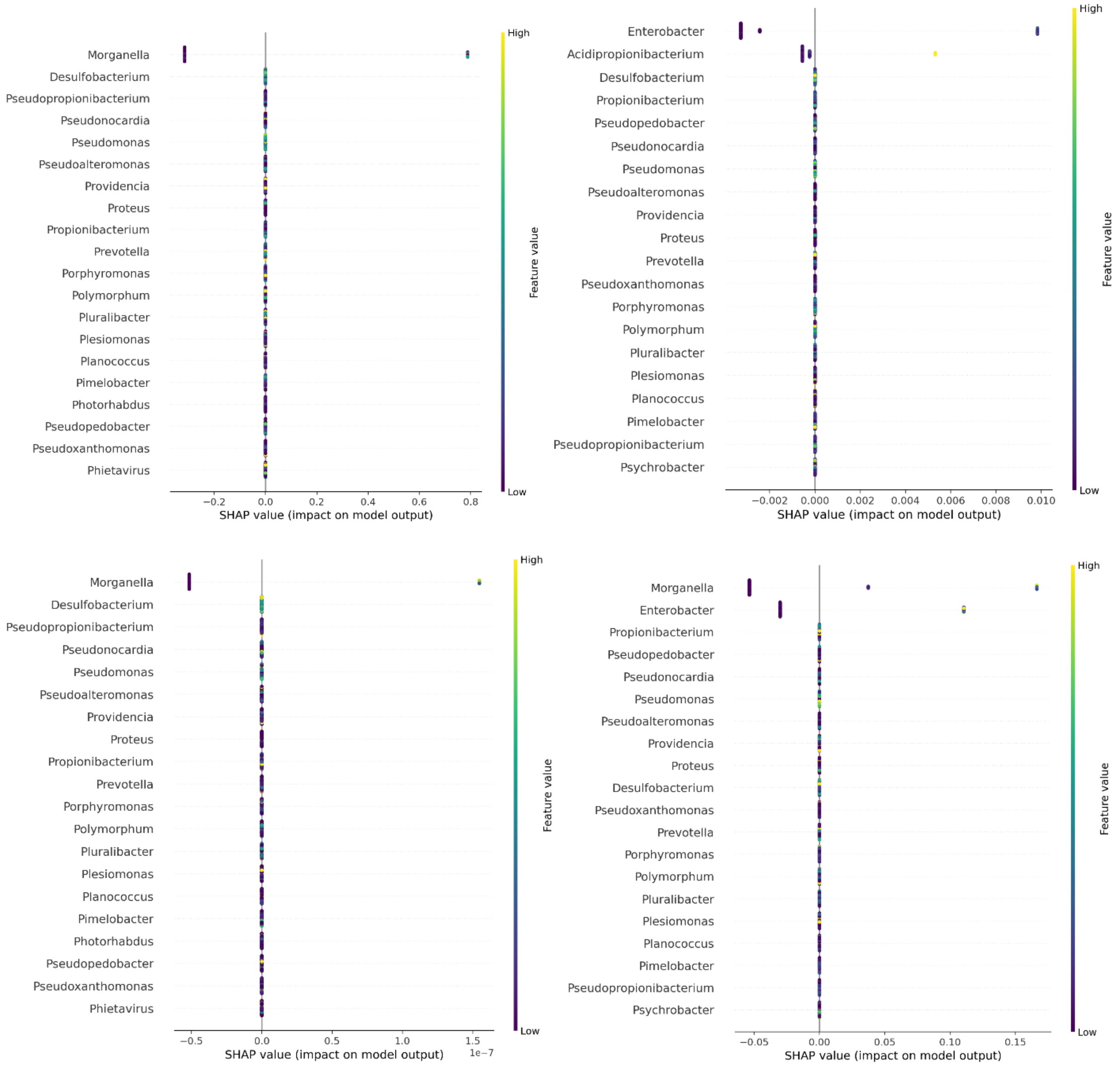

Supplemental Figure S4: SHAP results. SHAP dot plots for the most impactful features when predicting (a) Outside farm or (b) Antibiotic treated sample class vs. baseline in four independent iterations.
